# Supplementary figures and images for: Experimental emergence of conventions in human dyads
Source: PLoS One. 2026 Jul 27;21(7):e0341532. doi: 10.1371/journal.pone.0341532 (PMC13405092; doi:10.1371/journal.pone.0341532)

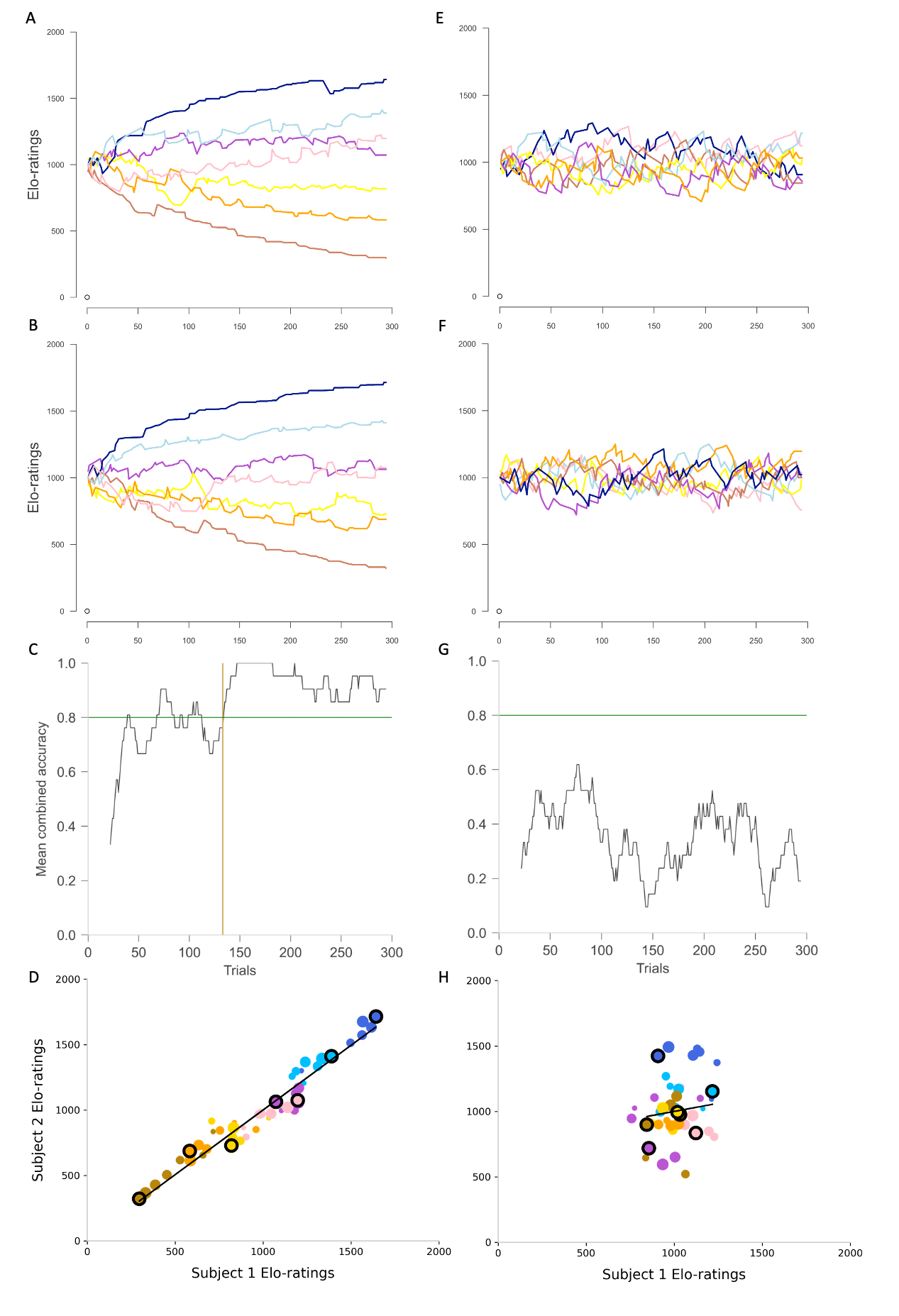

Supplement: S1 Fig — Left panels (A-D) show a pair with a convention; right panels (E-H) show a pair without a convention. (A) and (B) show evolution of Elo-ratings for Subject 1 and Subject 2, respectively, of an example pair that developed a convention. (C) Mean combined accuracy as a function of trial number, illustrating the emergence of a convention for an example pair. Combined accuracy is a metric that takes into account (i) whether the two subjects chose the same color and (ii) whether both subjects’ choices matched their respective color hierarchies developed up to that point. Mean combined accuracy is calculated over rolling windows of 21 trials. Note that the plot starts at trial 21 and each data point represents the mean of the previous 21 trials. Horizontal green line indicates threshold above which pairs had to maintain performance for at least 21 consecutive overlapping windows in order to pass criterion; vertical yellow line indicates the trial at which this pair was judged to have passed criterion, i.e., where a shared color hierarchy emerged and was maintained between the two subjects. (D) Elo-ratings of the two subjects plotted together across the session for every set of 42 trials (this included all possible color combinations being presented exactly twice). Increasing size of the circles correspond to progression through 7 such sets of 42 trials (yielding a total of 294). The final Elo-rating values at the end 294 trials are plotted with a black outline and the black line represents the regression through these points. (E)-(H) show the same plots for a pair without a convention. (TIF) [file pone.0341532.s001.tif]

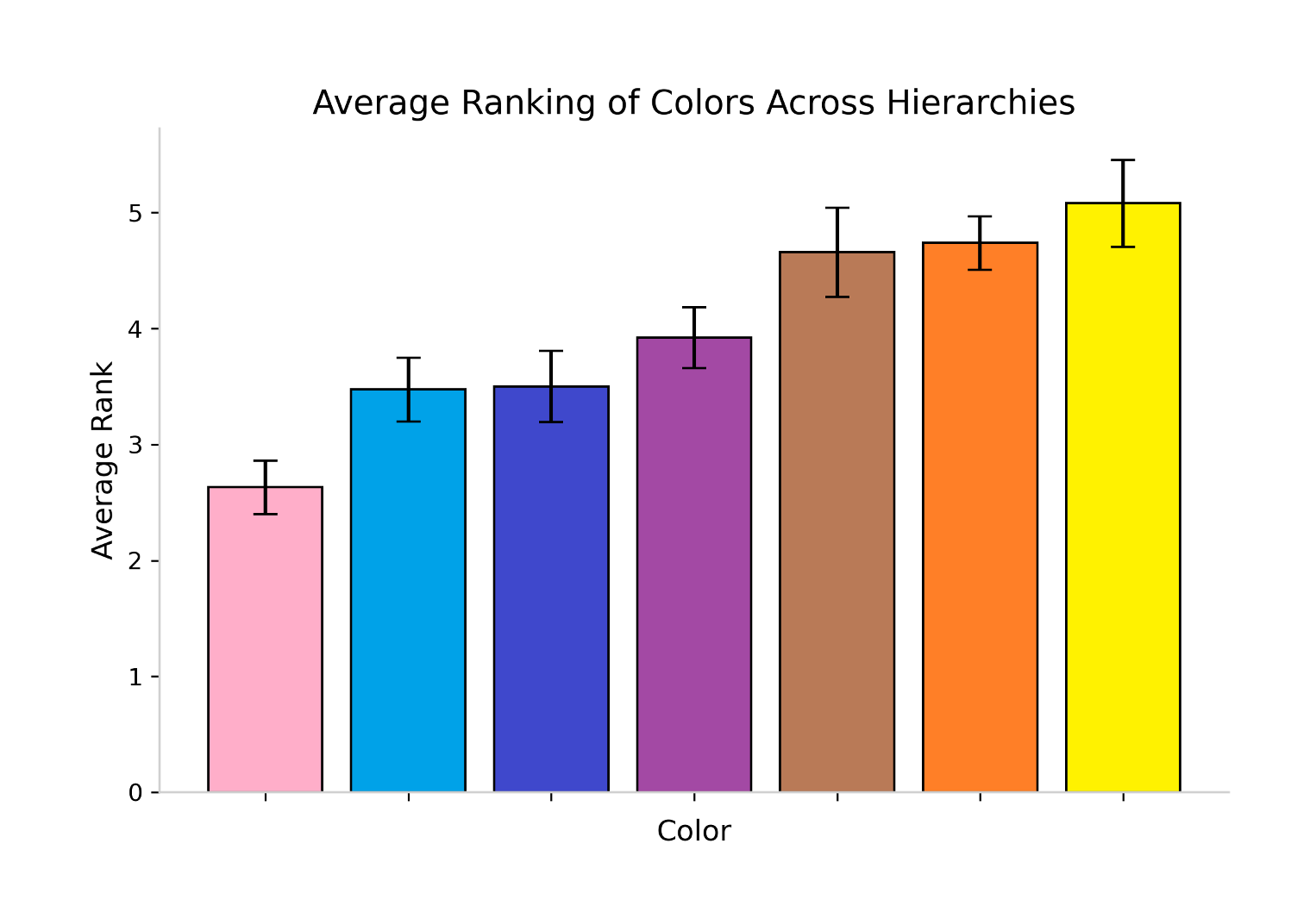

Supplement: S2 Fig — Lower rank number implies the color appeared at the top of more hierarchies. Error bars show SEM (standard error of the mean). (TIF) [file pone.0341532.s002.tif]

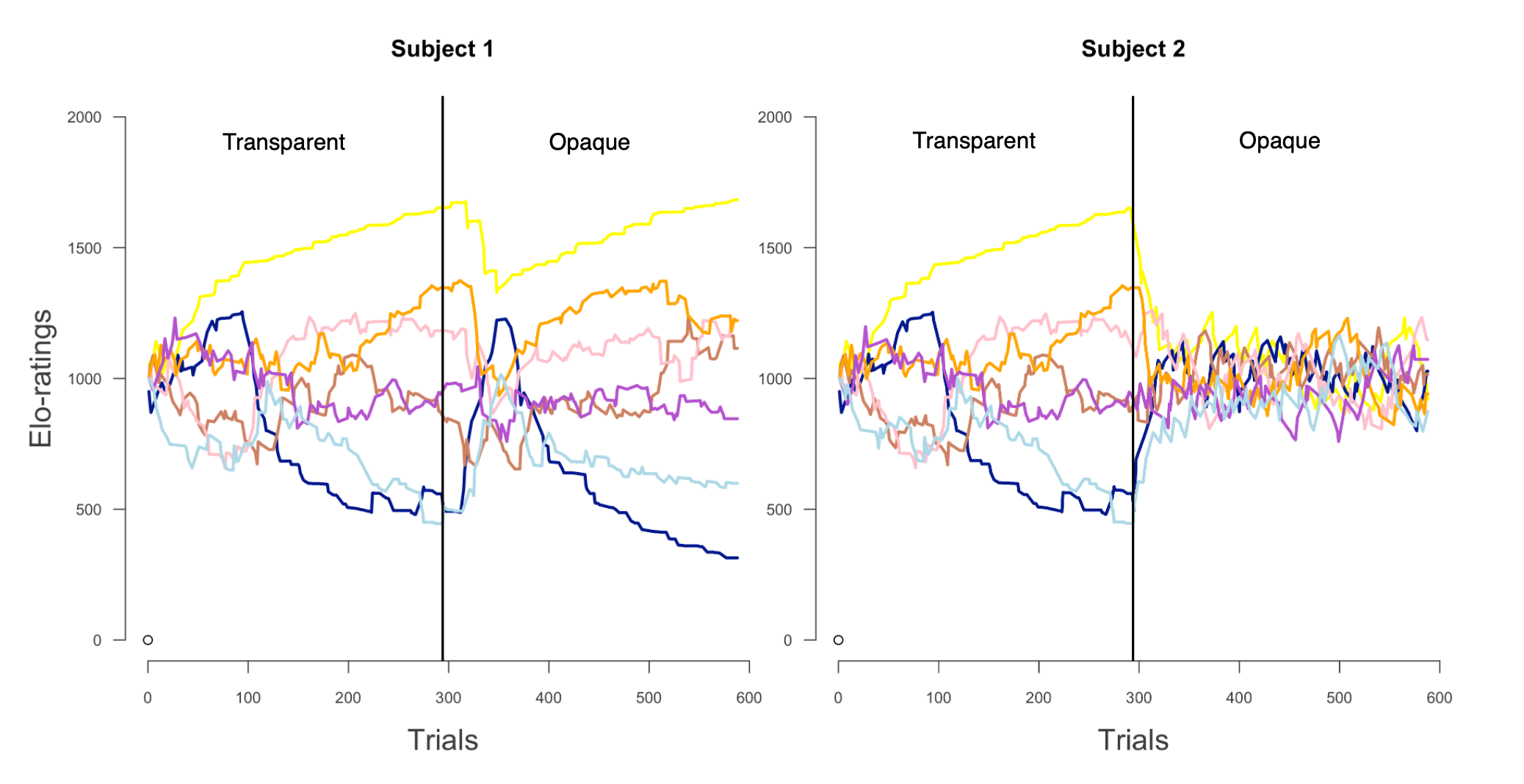

Supplement: S3 Fig — This example pair completed the first session in NI-T condition followed by an opaque session. Analysis of Session 1 (transparent) shows a convention emerging in the pair but Session 2 (opaque) data suggest that Subject 1 (left) may have been using a color hierarchy to solve this task while Subject 2 (right) was likely copying Subject 1 in Session 1. What appeared to be a convention in the transparent session disappeared in the following opaque session. (TIF) [file pone.0341532.s003.tif]
