# Supplementary material for: Experimental emergence of conventions in human dyads
Source: PLoS One. 2026 Jul 27;21(7):e0341532. doi: 10.1371/journal.pone.0341532 (PMC13405092; doi:10.1371/journal.pone.0341532)
Supplement: S1 Table — (DOCX) [file pone.0341532.s005.docx]

| Condition | Number of subjects | Age  M (SD) | Female | Male |
| --- | --- | --- | --- | --- |
| I-O | 20 | 20.45 (2.44) | 17 | 3 |
| I-T | 20 | 20.45 (3.32) | 16 | 4 |
| NI-O | 20 | 20.10 (2.45) | 17 | 3 |
| NI-T | 20 | 20.20 (2.19) | 17 | 3 |
