# Supplementary material for: Experimental emergence of conventions in human dyads
Source: PLoS One. 2026 Jul 27;21(7):e0341532. doi: 10.1371/journal.pone.0341532 (PMC13405092; doi:10.1371/journal.pone.0341532)
Supplement: S2 Table — (DOCX) [file pone.0341532.s006.docx]

| Level | Examples (hypothetical) | Examples (from subjects) |
| --- | --- | --- |
| 0 | 1. I clicked the right square 2. I picked my favourite colours | 1. “always clicking the left square” 2. “I chose the less vibrant/lighter color out of the two colors.” |
| 1 | 1. I picked the colours my partner seemed to picking 2. I tried to follow my partner’s colour preferences | 1. “I tried to guess what my partner will choose based on her choice from last round” 2. “I memorized which color the other person preferred for each specific pair” |
| 2 | 1. I always chose yellow because I think that’s what my partner expected me to choose based on our past choices 2. I would change my choices if I felt like my partner was not expecting one over the other | 1. “follow a pattern and try to be as predictable as possible” 2. “Trying to predict the preferences of the other participant and trying to be consistent in my preferences of color” |
