# Supplementary material for: Experimental emergence of conventions in human dyads
Source: PLoS One. 2026 Jul 27;21(7):e0341532. doi: 10.1371/journal.pone.0341532 (PMC13405092; doi:10.1371/journal.pone.0341532)
Supplement: Attachment — Submitted filename: Referee_Report_PLOS One.pdf [file pone.0341532.s007.pdf]

Referee Report for "Experimental Emergence of Conventions in Human Dyads"

Recommendation

I recommend minor revisions to this paper.

Comments

This paper uses a human subjects experiment to examine the emergence of convention addressing the first feedback loop (between the individual and the dyad) by varying subjects' prior knowledge, visual access, and prior experience. The results add to theoretical knowledge of how different mechanisms may interact to yield solutions to novel coordination problems in dyads and how these solutions may be transmitted and diffuse through larger populations.

Related Literature

1. The topic of this paper is Convention, a term easily confused with other social terms (moral or social norms, etc.). It would be better if you could briefly explain why you focus on Convention and what distinguishes it from those terms. Below is the Taxonomy of Norms from Bicchieri (2005) "*Grammar of Society (2005)*", as an example.

<table>
<tr><td></td><td></td><td colspan="2">Conditional Preferences?</td><td></td><td></td></tr>
<tr><td></td><td></td><td>No</td><td>Yes</td><td></td><td></td></tr>
<tr><td rowspan="3">Mutual Expectations?</td><td>No</td><td>Personal Norm</td><td>Descriptive Norm</td><td></td><td rowspan="3">Normative Expectations?</td></tr>
<tr><td rowspan="2">Yes</td><td rowspan="2">Moral Norm</td><td>Convention</td><td>No</td></tr>
<tr><td>Social Norm</td><td>Yes</td></tr>
</table>

Table 1: Bicchieri (2005): Taxonomy of Norms

2. Here are some other related papers that may also be helpful:

    - Freire et al. (2020) " Modeling the formation of social conventions from embodied real-time interactions," *PloS one* 15(6), e0234434.
    - Przepiorka & de Kwaadsteniet (2021) "The emergence of conventions in the repeated volunteer's dilemma: The role of social value orientation, payoff asymmetries and focal points," *Social Science Research* 93, 102488.
    - Hawkins et al. (2023) "From partners to populations: A hierarchical Bayesian account of coordination and convention," *Psychological Review* 130(4):977.

Experimental Design

1. If you were using ORSEE to recruit the human subjects, cite Greiner, B. (2015) "Subject pool recruitment procedures: organizing experiments with ORSEE," *Journal of the Economic Science Association* 1(1), 114-125. Otherwise, briefly explain how you recruit subjects to ensure randomness.

2. Please include a balance test showing that the 101 subjects' characteristics were balanced across treatments.

    - Were the subjects (especially graduate students) majored in economics? If so, please check the proportion of them (though I don't think there were many), since they may have better knowledge of game theory and social dilemmas.
    - Add a footnote that 21 out of the 101 subjects participated in both Phases, while the rest of them only participated in one Phase. Make it a diagram or a short table if needed.

3. There's no partition in Figure 1A, while it should be (may be lower than that in Figure 1B). Update Figure 1A as much as possible so readers can tell that the subjects cannot see their counterparts in person but can see the screen.

4. I'm a bit curious about (i) whether the order of color display (left-right) affects the frequency with which a subject chooses a particular color; (ii) whether the formed hierarchies mostly follow the order of the rainbow (treating brown as red).

5. What's the time lag between Phases I and II? Can the experienced subject perfectly recall the color hierarchy? What if there's a tie in the previous color hierarchy? Do you allow ties in the recall process?

6. Have you collected any information on subjects' personal characteristics, such as selfish/altruism, social value orientation, or risk preferences? For pairs that differ in individual hierarchy, a pair of "dominance + follower" type is more likely to form a convention than a pair of "dominance + dominance".

7. (I may have missed this.) Please include the average cash payment and the conversion rate you used between experimental points and Dollars.

Pre-registration and Hypotheses

1. No information on pre-registration is provided in the draft. Please state where you have pre-registered your study (a link shall do) and whether the hypotheses and analysis plan are included in the pre-registration.

2. Reconstruct the Results part so that each finding can be linked to the corresponding hypothesis.

   - In the last paragraph of the Introduction, you mentioned *"We expected to see conventions emerge more often and faster when ..."*. Separate it into three hypotheses and insert them in the corresponding places, preferably after you introduced your experimental design. And, in the Results section, you can have a 1-sentence version of each finding, either at the beginning or the end of a subsection, to echo the corresponding hypothesis.

Minor comments

1. Be careful with the commas. Here are a few examples:

   - Section 1, second paragraph: *Following the seminal work of Lewis [7]*, *we refer to ...*
   - Section 2.1, first paragraph: *in Phase I*, *we ... while in Phase II*, *we explored ...*
   - Section 2.2, Figure 1 paragraph: *...in the (A) Transparent condition*, *where subjects ...*, *and (B) Opaque condition*, *where subjects ...*

2. Be consistent with the terminology you used. For example, whether the words have a hyphen in the middle.

3. In the Figure 1 paragraphs, please refer to A as Figure 1A, or use separate sentences, something like: *"C and D in Figure 1 demonstrate the screens of the correct and incorrect trials, respectively. If both subjects ... (Fig 1C); if ... (Fig 1D)."* Same as the Figure 2 paragraph.

4. Section 2.3, third paragraph: It should be 98 trials, not 98 sessions.
