# Supplementary material for: Experimental emergence of conventions in human dyads
Source: PLoS One. 2026 Jul 27;21(7):e0341532. doi: 10.1371/journal.pone.0341532 (PMC13405092; doi:10.1371/journal.pone.0341532)
Supplement: Attachment — Submitted filename: response_to_reviewers.pdf [file pone.0341532.s009.pdf]

Point-by-point responses to Editor's and Reviewers' comments "Experimental Emergence of Conventions in Human Dyads" by O. Mohan & D. Biro (PONE-D-26-01263)

(editor and reviewer comments in black, our responses in blue; line numbers refer to those in the 'clean' version)

Editor

Journal Requirements:

1. Please ensure that your manuscript meets PLOS ONE's style requirements, including those for file naming. The PLOS ONE style templates can be found at https://journals.plos.org/plosone/s/file?id=wjVg/PLOSOne_formatting_sample_main_body.pdf and https://journals.plos.org/plosone/s/file?id=ba62/PLOSOne_formatting_sample_title_authors_affiliations.pdf

Thank you – we have ensured that our manuscript follows PLOS ONE's style requirements.

2. We noted in your submission details that a portion of your manuscript may have been presented or published elsewhere. "An abstract of our study has appeared in the proceedings of the 46th Annual Meeting of the Cognitive Science Society (2024). This reports the results in broad terms, but no data or figures were included. Link: https://escholarship.org/uc/item/9284q9zc" Please clarify whether this [conference proceeding or publication] was peer-reviewed and formally published. If this work was previously peer-reviewed and published, in the cover letter please provide the reason that this work does not constitute dual publication and should be included in the current manuscript.

A 6-page paper was submitted to the 46th Annual Meeting of the Cognitive Science Society (2024). This was peer-reviewed and accepted to the conference as a poster. For the version included in the conference proceedings, we had the option to publish either only the abstract or the entirety of the 6-page paper. We chose to only publish the abstract and as such we believe the present manuscript does not constitute dual publication. We have also included this explanation in our cover letter.

3. If the reviewer comments include a recommendation to cite specific previously published works, please review and evaluate these publications to determine whether they are relevant and should be cited. There is no requirement to cite these works

unless the editor has indicated otherwise.

We confirm that we have thoroughly reviewed all new citations included in the revised manuscript on the reviewers' recommendation.

4. Please review your reference list to ensure that it is complete and correct. If you have cited papers that have been retracted, please include the rationale for doing so in the manuscript text, or remove these references and replace them with relevant current references. Any changes to the reference list should be mentioned in the rebuttal letter that accompanies your revised manuscript. If you need to cite a retracted article, indicate the article's retracted status in the References list and also include a citation and full reference for the retraction notice.

We confirm that all changes to the reference list are mentioned below, alongside the corresponding responses to reviewers' comments and suggestions. None of the papers we cite have been retracted.

Reviewer #1

Mohan & Biro present an experimental study of convention formation, manipulating visual access to the partner's screen, whether participants were informed about the reward structure (i.e., both selecting the same color), and testing how conventions from one pair can get passed onto a new generation.

I co-reviewed this paper together with one of my Postdocs, and we both found it to be a very enjoyable experience. The introduction was very well written and did a great job of clarifying terminology and in summarizing the related literature. The research question was also very well put, and the hypotheses were appropriate and justified, even if they didn't always work out.

We thank the reviewer and postdoc for this highly positive evaluation – we are pleased they both found the paper enjoyable!

Together, we have one suggestion for a theoretical explanation that could explain the results, one formatting suggestion that could improve readability, and then a small list of minor comments.

Theoretical explanation for why task information made it harder for conventions to form

One relevant theoretical perspective could be from Roberts & Goldstone (PLOS ONE 2011), where they show that having too much reactivity prevented groups from converging on a correct solution. Their task is framed as dinner guests figuring out how

much to contribute towards a bill, with feedback being binarized as correct vs. incorrect. Having too much reactivity in the group actually made it harder to reach the solution, since the group would over-correct and vacillate between too high and too little. A similar phenomena could also explain the current results, since both individuals being informed of the goals could be trying to signal a convention, leading to over-correction. Thus, in this dyadic setting, you might need a leader-follower dynamic for conventions to converge, with a more active leader and a more passive follower. Instead, the informed condition could lead to unstable k-level reasoning that ends up hindering more than helping.

Thank you for these detailed thoughts – they very closely mirror our interpretation! We fully agree that a form of "over-correction" or heightened reactivity may indeed have been a reason behind the delayed emergence of conventions in the Instruction condition. We tried to allude to this with the sentence starting on Line 640 but now see that this needed further elaboration. We have therefore expanded on this point along the lines you suggest, and to better explain our hypothesis we have included a brief summary of the suggested paper and how it aligns with our results (Lines 644-648).

Combining Methods:Analyses with results
If the journal style allows, the Methods: Analyses section could be joined with results in order to make the details closer to where they become relevant. Currently, the formatting places a large distance between the description of methods and the context in which they are applied, making it hard to assess the appropriateness and also hard to interpret the results (if details have been forgotten). Additionally, this current formatting also places Figure 2 quite far from the results in which it presents.

We agree that the details of the analyses were positioned far away from the results, which can make it difficult for readers to follow what approach was used to arrive at specific results. However, given that our analysis section is rather long, we were worried that merging it with results would cause the key findings to be "buried" in long sections of text. Instead, we have added 1-2 sentences at the start of each subsection in the Results, summarizing the main questions and analyses used before moving on to their conclusions – we hope this helps to better prepare the reader to understand each of our results in turn. In addition, we have split Figure 2 into new Figures 2 and 3 in order to keep the panels relevant to the methods close to where those methods are described, while moving the other panels closer to where the relevant results appear in the text.

Minor comments

- Goldstone & Ashpoole (2004) could be a good reference for early work on convention formation (~ pg. 3)

Thank you for this suggestion – we have now read this paper; however, we chose not to include it among our references as we did not feel it was directly relevant to the

definition of conventions we used.

- Line 69: Roger's paradox is probably not the best example in the context of conventions since it explores the hypothesis that culture emerged from natural origins

Thank you for raising this; we agree. This example has now been removed.

- Line 149: more consistency could be used when referring to the instruction type (before it was referred to as informed vs. uninformed)

We used the terms “informed” and “uninformed” only in our Introduction, and primarily in the context of discussing the emergence and maintenance of conventions at individual, group, and population levels (originally on Line 78, now on Line 91 in the revised ms) – this was not intended to refer to our experimental conditions but to broad questions one might raise about conventions more generally. However, we note we did also use these terms once in reference to our design (originally on Line 116), which we have now reworded (Line 130). Elsewhere in the manuscript we consistently refer to our treatments as “instruction” and “no instruction” (designed to simulate informed vs uninformed subjects). These terms are orthogonal to “experienced” and “naive” subjects (e.g. Line 132) which we use to refer to subjects in Phase II who had or had not participated in Phase I, respectively. (See also our response to Reviewer 1’s penultimate point for further discussion of the different ways in which our subjects could be described as “informed” or “uninformed” across both Phases I and II.)

- Lines 184-186: A subordinate clause would be appreciated to indicate what should be avoided by randomizing colors

We have added a clarification that randomizing locations was intended to prevent subjects using the location rather than the color of the squares as the basis for matching (Line 215).

- Could participants talk to one another during the 5 minute break?

No, they were not – they received specific instructions not to communicate at any point. This was mentioned in the original manuscript but we were not specific that this extended to breaks, so we have now included this detail (Line 190). We have also added a clause on Line 223-224 to remind readers.

- Were there different color sets across sessions? Could representations from one session bleed over to the next?

The same 7 colors were used in both sessions. This detail has been added to Lines 225-226 in the revised ms.

- How long was the gap between Phase I and Phase II? Could there be difficulty in remembering the conventions from Phase I?

The gap between the two phases ranged from a minimum of 42 days to a maximum of 126 days (Mean = 71.5, SD = 16.77 days) – these are indeed rather long delays and by prompting subjects to recall their Phase I hierarchies at the start of Phase II we were able to show that they did not remember the precise hierarchies accurately (see Fig. 4C, correlation between “Experienced Phase I” and “Experienced recall”). We therefore compared both the Phase I and the recalled hierarchies to those that emerged during Phase II. We have added a clarification to the Analysis section of Phase II (Lines 437-439).

- Figure 2 could benefit from titles to make panels A-C more self-evident. The current placement in the methods section also separates it quite a bit from the results section when it becomes relevant

We are hesitant to give brief titles (e.g., clear hierarchy, no hierarchy, etc.) to panels A-C because we do not want these to seem like explicit categories that individual color hierarchies fall into and would prefer to represent them as examples from a continuum. That being said, we agree that the placement is not ideal, so we have split Figure 2 into two different figures. The new Figure 2 only consists of the example color hierarchies which are helpful in visualizing and describing the data on which many of our analyses are based. The rest of the figure is now Figure 3, which appears later in the manuscript and showcases various results, including those from two new analyses added in response to your suggestions (see next two comments: overall performance and reaction time).

- Overall performance from Sec 3.1 could be added as a plot

Thank you for this suggestion; we agree and have added an overall performance plot as the new Figure 3A.

- While the current reaction time results didn’t work, what about testing within-dyad differences for pre vs. post convention formation? I suspect that this would grant greater power for such an analysis, although the effect of trial number would also have to be controlled for

Thank you very much for this great suggestion. We have executed this analysis as a mixed-effects model, to control for trial number, and observed an effect of pre- vs. post-

convention on reaction time for the pairs that had a convention, but only in NI-O, I-T and NI-T. We have included this information in the manuscript (detailed description of the model in the Analysis section on Lines 278-289 and the corresponding results in the Results section on Lines 461-464-457) along with a figure showing the raw reaction time data (Fig. 3B).

- The justification to define stability of conventions emerging with at least 100 trials to analyze seems rather arbitrary

The decision to calculate stability for 100 trials after emergence was done to ensure a fair comparison across pairs from the different conditions. In other words, we wanted to standardize this measure, since it is sensitive to the length of window analyzed. Our choice of 100 trials was indeed arbitrary, but it was chosen on the basis of pilot data to ensure a sufficiently long window to calculate stability meaningfully. In our main dataset, there were only two pairs that did not have sufficient trials post-convention (23 and 87 trials, both in the I-O condition) and were thus not included in the stability analysis. To ensure that this did not influence the results, we have reanalyzed the data with all post-convention trials included for all pairs (i.e. with no pairs removed): these are now plotted as a new Supplementary Figure (Fig. S4). The results of the corresponding analyses are reported in the caption to Figure S4 and are referenced in the main text on Lines 505-507 for Phase I and Lines 577-578 for Phase II, confirming that our results hold even if this alternative approach had been chosen. We have also added extra explanation in the methods to clarify our approach (Lines 377-379).

- Line 410: n = 19
- Line 411: n = 21

Thank you for pointing these out; they have been corrected (Lines 460 and 461).

- Lines 545-551: Could this be explained by the arbitrariness of the task (i.e., is building a model about something arbitrarily debilitating)?

We are not sure we understand what is meant by arbitrariness in this context. If it is about the solution (the actual color hierarchy being used) then this arbitrariness is common across both I and NI. If the arbitrariness is referring to trying to build a model about the task without knowing what exactly the goal is (the NI conditions) then our results show the opposite of this; it seems subjects more readily sample the "environment" (which in this case is the task and the partner's actions) and build models when the task is *more* arbitrary (NI conditions).

- Aplin et al., (Nature 2015) could also be a good reference for conventions in non-

human animals, where they experimentally induced conventions in great tits that persisted through cultural transmission

Based on Reviewer 2’s suggestion, we have added text to the Introduction (Lines 49-59) to distinguish between norms and conventions. We have also referenced some reviews that discuss non-human animal norms and conventions. However, we want to refrain from using examples like Aplin et al., 2015 since the terms norms and conventions are not used consistently in the literature (for example, according to the definition we adopt, the birds in Aplin et al. 2015 are not using conventions since there is no multi-agent coordination involved). Nonetheless, on the basis of this suggestion we have included some additional review papers that address this issue (Andrews et al. 2024, Stephens & Heinen, 2018; Line 59) in order to provide interested readers with further material on the topic.

-Lines 668-669: This could be understood as a mixed condition (I-NI). What could be the reason for the high speed of emerging conventions under this mixed condition, given the argument that instructions slowed down the emergence of conventions in Phase I?

Indeed, Phase II can be thought of as a mixed condition in the sense that the naïve individual has less “information” than the experienced individual. Crucially, however, it is the naïve subject that is the strict equivalent of the I condition from Phase I (and there is no NI player in Phase II). The experienced player in fact has *more* information than any subject was provided with in Phase I by virtue of already knowing the optimal solution for solving the task in the opaque condition (must stick to consistent color hierarchy). Our intention in referring to the two subjects in Phase II as experienced and naïve was to highlight this information differential and distinguish it from that created by providing subjects with instructions vs no instructions in Phase I. In turn, our results suggest that knowledge of the optimal solution from the start by one of the players enhances the pair’s ability to converge on a color hierarchy quickly. We then hypothesize that this knowledge possessed by the experienced player could have facilitated this rapid convergence in one of two ways: the experienced subject could have been unwavering in their choices in order to steer the pair towards their recalled hierarchy or they could have adapted more readily to their partner’s preferences to cement a new color hierarchy. Based on comparisons of the recalled hierarchies with those that Phase II pairs converged on (Fig. 4C), we suggest that the latter process was more likely. These points are now clarified further on Lines 779-780.

- The know-how vs. know-what section in the discussion is currently missing references. Maybe Bandini et al., (2020) is a relevant reference in the context of social/cultural learning, but there are likely to be earlier uses of the term

Thank you for bringing this to our attention. We agree and have added the above suggested reference as well as another, earlier reference (Legare 2017) to the discussion of know-how vs. know-what (Line 785).

Reviewer #2

Recommendation

I recommend minor revisions to this paper.

Comments

This paper uses a human subjects experiment to examine the emergence of convention addressing the first feedback loop (between the individual and the dyad) by varying subjects' prior knowledge, visual access, and prior experience. The results add to theoretical knowledge of how different mechanisms may interact to yield solutions to novel coordination problems in dyads and how these solutions may be transmitted and diffuse through larger populations.

We thank Reviewer 2 for their kind evaluation and recommended minor revisions.

Related Literature

1. The topic of this paper is Convention, a term easily confused with other social terms (moral or social norms, etc.). It would be better if you could briefly explain why you focus on Convention and what distinguishes it from those terms. Below is the Taxonomy of Norms from Bicchieri (2005) "Grammar of Society (2005)", as an example.

<table>
<tr><th></th><th></th><th colspan="2">Conditional Preferences?</th><th></th><th></th></tr>
<tr><td></td><td></td><td>No</td><td>Yes</td><td></td><td></td></tr>
<tr><td rowspan="3">Mutual Expectations?</td><td>No</td><td>Personal Norm</td><td>Descriptive Norm</td><td></td><td></td></tr>
<tr><td rowspan="2">Yes</td><td rowspan="2">Moral Norm</td><td>Convention</td><td>No</td><td rowspan="2">Normative Expectations?</td></tr>
<tr><td>Social Norm</td><td>Yes</td></tr>
</table>

Table 1: Bicchieri (2005): Taxonomy of Norms

Thank you for this suggestion – we agree that additional discussion of terminology would be helpful to orient readers, especially given the non-standardized use of terms such as "norms" and "conventions" in the literature (see also our response to Reviewer 1's third-from-last comment that illustrates this point). We have added a paragraph near

the beginning of the Introduction (Lines 49-59) to briefly explain how researchers have attempted to distinguish between norms and conventions, as well as to clarify our own usage. We thank Reviewer 2 for suggesting the Biccheri 2005 work, which has helped us with criteria for classification and which we have added as a new citation.

2. Here are some other related papers that may also be helpful:
• Freire et al. (2020) “ Modeling the formation of social conventions from embodied real-time interactions,” PloS one 15(6), e0234434.
• Przepiorka & de Kwaadsteniet (2021) “The emergence of conventions in the repeated volunteer’s dilemma: The role of social value orientation, payoff asymmetries and focal points,” Social Science Research 93, 102488.
• Hawkins et al. (2023) “From partners to populations: A hierarchical Bayesian account of coordination and convention,” Psychological Review 130(4):977.

Thank you for these recommendations. We have added two of these suggested papers (Freire et al. 2020 and Hawkins et al. 2023; Lines 78-79) as references in the Introduction where we discuss computational models of conventions.

Experimental Design

1. If you were using ORSEE to recruit the human subjects, cite Greiner, B. (2015) “Subject
pool recruitment procedures: organizing experiments with ORSEE,” Journal of the Economic Science Association 1(1), 114-125. Otherwise, briefly explain how you recruit subjects to ensure randomness.

We did not use ORSEE – we recruited subjects using flyers placed around the University of Rochester campus. They were asked to fill out a form with availabilities if they were interested in participating in our study and were assigned into pairs based on availability. We have added this detail in Methods: Subjects and experimental procedures (Lines 159-160).

2. Please include a balance test showing that the 101 subjects’ characteristics were balanced across treatments.

The only demographic information we collected from our subjects was age and sex. We have run balance tests on these data and included the table below with raw data as new supplementary material (Supplementary Table 1, accompanying text on Lines 187).
Results of the balance tests:
Age: ANOVA $F(3, 76) = 0.09$, $p = 0.965$
Sex: Chi-Squared test $\chi^2(3) = 0.28$, $p = 0.965$

Condition	N	Age M (SD)	Female	Male
I-O	20	20.45 (2.44)	17	3
I-T	20	20.45 (3.32)	16	4
NI-O	20	20.10 (2.45)	17	3
NI-T	20	20.20 (2.19)	17	3

• Were the subjects (especially graduate students) majored in economics? If so, please check the proportion of them (though I don't think there were many), since they may have better knowledge of game theory and social dilemmas.

Thank you for this interesting suggestion. Unfortunately, we did not collect this information from our subjects and hence cannot report proportions. Nonetheless, since we advertised the study widely on campus (and as a generic "decision-making task", i.e., without it being obvious that game theoretic elements were involved), we do not have reason to believe that economics majors were disproportionally represented in our sample.

• Add a footnote that 21 out of the 101 subjects participated in both Phases, while the rest of them only participated in one Phase. Make it a diagram or a short table if needed.

We have added this detail Methods: Subjects and experimental procedures (Line 163).

3. There's no partition in Figure 1A, while it should be (may be lower than that in Figure 1B). Update Figure 1A as much as possible so readers can tell that the subjects cannot see their counterparts in person but can see the screen.

Thank you bringing this to our attention. We have updated Figure 1A and 1B with AI generated images that visualize the set-up in 3D and hopefully provide more clarity on the Transparent vs Opaque conditions.

4. I'm a bit curious about (i) whether the order of color display (left-right) affects the frequency with which a subject chooses a particular color; (ii) whether the formed hierarchies mostly follow the order of the rainbow (treating brown as red).

These are also interesting suggestions, and we have now looked into the relevant patterns in more detail and report them in the manuscript.
(i) The left-right order of the color display was randomized across participants, although some subjects (especially in the transparent conditions) reported always clicking left or right as a strategy. We did run an analysis after reading this comment to calculate the number of colors (out of 7 total) that were significantly more likely to be clicked when appearing on one side over the other for each subject. The median number of colors in

this calculation across all our subjects was 0.5 colors. Therefore, we do not believe there was an effect of the order or position (left-right) of the color display on the frequency with which a given color was chosen. However, we did find that running this analysis allowed us to more clearly differentiate potential leader-follower dynamics in pairs in the transparent condition. For example, in one pair from the I-T condition, one subject always clicked left while the other had roughly 50% chance of clicking left. From this, we can infer that the former was likely to be the “leader” (i.e., the subject who clicked first) and the latter was the “follower” (the subject who then matched the partner’s choice). We thank the reviewer for bringing this to our attention and we have added this observation to the relevant part of the Discussion (Lines 707-710).
(ii) We ran an analysis of the average rank of the seven colors across all the conventions that emerged to check whether there was any universality in the hierarchies formed. This analysis is reported in Supplementary Figure S1. While we observed a broad pattern (from most preferred to least preferred: pink, light blue, dark blue, purple, brown, orange, yellow), there was no overall significant agreement across all subjects that had developed a color hierarchy convention. From subjects’ self-reported strategies, it appeared that they relied mostly on their subjective liking of specific colors.

5. What’s the time lag between Phases I and II? Can the experienced subject perfectly recall the color hierarchy? What if there’s a tie in the previous color hierarchy? Do you allow ties in the recall process?

The gap between the two phases ranged from a minimum of 42 days to a maximum of 126 days (Mean = 71.5, SD = 16.77 days); this has now been added to the Analysis section of Phase II (Lines 437-439). These were long gaps, and our analysis (Fig. 4C) shows that the subjects indeed could not recall their previously established hierarchies perfectly. Hence, we primarily used the recalled hierarchy at the start of Phase II as a measure of the extent to which specific hierarchies were transmitted to the naïve partner at the end of Phase II. We did not allow for ties in the recalled hierarchy (our interpretation of a tie would be subjects’ uncertainty about relative ranks in a specific pairing of colors rather than a true tie, where ties are not expected to be stable in the long run since they do not reliably provide a solution in our task).

6. Have you collected any information on subjects’ personal characteristics, such as selfish/altruism, social value orientation, or risk preferences? For pairs that differ in individual hierarchy, a pair of “dominance + follower” type is more likely to form a convention than a pair of “dominance + dominance”.

We did not collect any information on personal characteristics, although we did look at the self-reported strategies to see if we could classify subjects as either leaders or followers in the transparent conditions. But since such data were sparse (most subjects did not spontaneously comment on this aspect) and somewhat subjective, we did not

run any further analysis. However, we do believe it would be useful to collect such information in future work and thank the reviewer for the suggestion.

On a related note, we fully agree with the point that certain kinds of asymmetries between subjects in a pair may facilitate the emergence of conventional solutions by short-circuiting overcorrection/oscillations after unsuccessful trials. For example, a pairing between a ‘stubborn’ individual (who is unwilling to adjust their preference) and a ‘flexible’ individual (who reverses their preference after an unsuccessful trial) would fare better than a pair of two flexible individuals: the latter would be more likely to be trapped in an oscillatory back-and-forth. This point is now included in the Discussion (Lines 652-653).

7. (I may have missed this.) Please include the average cash payment and the conversion rate you used between experimental points and Dollars.

All subjects were paid $10 per visit regardless of performance on the task. We have added this detail to the Methods (Lines 164).

Pre-registration and Hypotheses

1. No information on pre-registration is provided in the draft. Please state where you have pre-registered your study (a link shall do) and whether the hypotheses and analysis plan are included in the pre-registration.

We did not pre-register this study.

2. Reconstruct the Results part so that each finding can be linked to the corresponding hypothesis.
• In the last paragraph of the Introduction, you mentioned “We expected to see conventions emerge more often and faster when ...”. Separate it into three hypotheses and insert them in the corresponding places, preferably after you introduced your experimental design. And, in the Results section, you can have a 1-sentence version of each finding, either at the beginning or the end of a subsection, to echo the corresponding hypothesis.

Thank you for this suggestion – we agree the end of our Introduction could be strengthened by more clearly linking our experimental design to specific hypotheses. We have revised the final paragraph of the Introduction to implement the clarifications (Lines 130-148). We have also added one or two sentences to the start of each subsection in the Results, reiterating the analyses (in response to one of Reviewer 1’s suggestions) as well as the specific hypotheses being tested.

Minor comments

1. Be careful with the commas. Here are a few examples:

• Section 1, second paragraph: Following the seminal work of Lewis [7], we refer to ...
• Section 2.1, first paragraph: in Phase I, we ... while in Phase II, we explored ...
• Section 2.2, Figure 1 paragraph: ...in the (A) Transparent condition, where subjects ..., and (B) Opaque condition, where subjects ...

Thank you, we have corrected all three examples listed above and have gone through the manuscript and made several similar corrections where appropriate.

2. Be consistent with the terminology you used. For example, whether the words have a hyphen in the middle.

We have checked through the manuscript for inconsistent hyphenating and corrected several occurrences of missing or superfluous hyphens (e.g., color-hierarchy (adj.) vs color hierarchy (noun), color-pairing vs color pairing, inter-quartile vs interquartile, individual-level (adj.) vs individual level (noun)).

3. In the Figure 1 paragraphs, please refer to A as Figure 1A, or use separate sentences, something like: "C and D in Figure 1 demonstrate the screens of the correct and incorrect trials, respectively. If both subjects ... (Fig 1C); if ... (Fig 1D)." Same as the Figure 2 paragraph.

We believe this comment relates to the caption for Figure 1, rather than to references to its various panels in the main text. As such, we believe no changes are required as within a figure legend it is appropriate to refer to the panels by letter only. In the main text, Figure 1 panels are referenced as the reviewer suggests (see paragraphs above and below the figure caption).

4. Section 2.3, third paragraph: It should be 98 trials, not 98 sessions

Thank you for pointing out this error; it has been corrected.
