# Supplementary material for: Experimental emergence of conventions in human dyads
Source: PLoS One. 2026 Jul 27;21(7):e0341532. doi: 10.1371/journal.pone.0341532 (PMC13405092; doi:10.1371/journal.pone.0341532)
Supplement: Attachment — Submitted filename: response_to_reviewers_2.docx [file pone.0341532.s010.docx]

Point-by-point responses to Editor’s and Reviewers’ comments

“Experimental Emergence of Conventions in Human Dyads” by O. Mohan & D. Biro (PONE-D-26-01263R1)

(editor and reviewer comments in black, our responses in blue; line numbers refer to those in the ‘clean’ version)

Reviewer #1: I am grateful to the authors for addressing all of the previous comments in a thorough fashion. I have two very minor points I think can be addressed without needing to go through another round of review, which I describe below.

Thank you very much – we are pleased to hear that the Reviewer 1 was satisfied with our revisions.

Line 126 "Open questions“: Among other things, the authors find that a) more information leads to slower convention formation probably due to over-correction from social feedback (line 667), and b) visual access leads to less stability of conventions most likely due to higher level reasoning (line 710). Given these results, is perhaps a more successful strategy to learn reward-contingencies rather than to view the task as a coordination game? The authors hint at this possibility in lines 679-700 by referring to similar interpretations from other studies. But since this is the opening question of the paper, this alternative interpretation could be highlighted more in the paper.

Over-correction being the underlying cause of slower convention formation was only a hypothesis (and in fact is not supported by our analysis of self-reported strategies). However, we agree with the reviewer that the idea could be highlighted further. We did so in the Discussion by suggesting an extension to our task that could specifically test this hypothesis (lines 669-672).

Lines 563-575: The authors correctly hypothesized that conventions formed in the opaque condition would be more stable. As a measure of stability they calculated the maintenance of Elo-ratings across 100 trials. However, stability could also be interpreted as a convention being stable after a change. The unexpected maintenance of conventions formed during the opaque condition (Session 1) after switching to the transparent condition (Session 2) could thus be interpreted as another indicator of stability.

Thank you for pointing this out. We agree with the reviewer's comment and have updated the second paragraph of the Discussion to highlight how both stability within a session and maintenance across contexts serve as evidence for the self-sustaining nature of the shared color hierarchy as a convention (lines 631, 633–634).

Reviewer #2: I thank the author for addressing all comments carefully and professionally. I recommend accepting this paper. Green light now. Good job!

Thank you! We are glad to hear that we have addressed Reviewer 2’s comments in full.
