# Supplementary material for: Experimental emergence of conventions in human dyads
Source: PLoS One. 2026 Jul 27;21(7):e0341532. doi: 10.1371/journal.pone.0341532 (PMC13405092; doi:10.1371/journal.pone.0341532)
Supplement: Peer review history

# Peer review: Decision Letter 0

**DOI:** 10.1371/journal.pone.0341532.r001

**Authors:** Caleb Cox

25 Feb 2026

PONE-D-26-01263

Experimental Emergence of Conventions in Human Dyads

PLOS One

Dear Dr. Mohan,

Thank you for submitting your manuscript to PLOS ONE. After careful consideration, we feel that it has merit but does not fully meet PLOS ONE’s publication criteria as it currently stands. Therefore, we invite you to submit a revised version of the manuscript that addresses the points raised during the review process.

I have received reports from two reviewers. Both are positive, and recommend minor revisions. Reviewer 1 suggests a theoretical explanation for your results, along with some additional minor points. Reviewer 2 suggests some additional links to the literature and asks for information about pre-registration. It is okay if the study was not pre-registered, but you can simply clarify this point either way. With your revision, please include a response letter explaining how you address each point.

Please submit your revised manuscript by Apr 11 2026 11:59PM. If you will need more time than this to complete your revisions, please reply to this message or contact the journal office at plosone@plos.org. When you're ready to submit your revision, log on to https://www.editorialmanager.com/pone/ and select the 'Submissions Needing Revision' folder to locate your manuscript file.

Please include the following items when submitting your revised manuscript:

- A letter that responds to each point raised by the academic editor and reviewer(s). You should upload this letter as a separate file labeled 'Response to Reviewers'.
- A marked-up copy of your manuscript that highlights changes made to the original version. You should upload this as a separate file labeled 'Revised Manuscript with Track Changes'.
- An unmarked version of your revised paper without tracked changes. You should upload this as a separate file labeled 'Manuscript'.

-->If you would like to make changes to your financial disclosure, please include your updated statement in your cover letter. Guidelines for resubmitting your figure files are available below the reviewer comments at the end of this letter.

If applicable, we recommend that you deposit your laboratory protocols in protocols.io to enhance the reproducibility of your results. Protocols.io assigns your protocol its own identifier (DOI) so that it can be cited independently in the future. For instructions see: https://journals.plos.org/plosone/s/submission-guidelines#loc-laboratory-protocols. Additionally, PLOS ONE offers an option for publishing peer-reviewed Lab Protocol articles, which describe protocols hosted on protocols.io. Read more information on sharing protocols at https://plos.org/protocols?utm_medium=editorial-email&utm_source=authorletters&utm_campaign=protocols.

We look forward to receiving your revised manuscript.

Kind regards,

Caleb Cox

Academic Editor

PLOS One

Journal Requirements:

1. Please ensure that your manuscript meets PLOS ONE’s style requirements, including those for file naming. The PLOS ONE style templates can be found at

https://journals.plos.org/plosone/s/file?id=wjVg/PLOSOne_formatting_sample_main_body.pdf and

https://journals.plos.org/plosone/s/file?id=ba62/PLOSOne_formatting_sample_title_authors_affiliations.pdf

2. We noted in your submission details that a portion of your manuscript may have been presented or published elsewhere. “An abstract of our study has appeared in the proceedings of the 46th Annual Meeting of the Cognitive Science Society (2024). This reports the results in broad terms, but no data or figures were included. Link: https://escholarship.org/uc/item/9284q9zc” Please clarify whether this [conference proceeding or publication] was peer-reviewed and formally published. If this work was previously peer-reviewed and published, in the cover letter please provide the reason that this work does not constitute dual publication and should be included in the current manuscript.

3. If the reviewer comments include a recommendation to cite specific previously published works, please review and evaluate these publications to determine whether they are relevant and should be cited. There is no requirement to cite these works unless the editor has indicated otherwise.

4. Please review your reference list to ensure that it is complete and correct. If you have cited papers that have been retracted, please include the rationale for doing so in the manuscript text, or remove these references and replace them with relevant current references. Any changes to the reference list should be mentioned in the rebuttal letter that accompanies your revised manuscript. If you need to cite a retracted article, indicate the article’s retracted status in the References list and also include a citation and full reference for the retraction notice.

[Note: HTML markup is below. Please do not edit.]

Reviewers' comments:

Reviewer’s Responses to Questions

**Comments to the Author**

1. Is the manuscript technically sound, and do the data support the conclusions?

The manuscript must describe a technically sound piece of scientific research with data that supports the conclusions. Experiments must have been conducted rigorously, with appropriate controls, replication, and sample sizes. The conclusions must be drawn appropriately based on the data presented.

Reviewer #1: Yes

Reviewer #2: Yes

**********

2. Has the statistical analysis been performed appropriately and rigorously?

Reviewer #1: Yes

Reviewer #2: Yes

**********

3. Have the authors made all data underlying the findings in their manuscript fully available?

The PLOS Data policy requires authors to make all data underlying the findings described in their manuscript fully available without restriction, with rare exception (please refer to the Data Availability Statement in the manuscript PDF file). The data should be provided as part of the manuscript or its supporting information, or deposited to a public repository. For example, in addition to summary statistics, the data points behind means, medians and variance measures should be available. If there are restrictions on publicly sharing data—e.g. participant privacy or use of data from a third party—those must be specified.

Reviewer #1: No

Reviewer #2: Yes

**********

4. Is the manuscript presented in an intelligible fashion and written in standard English?

PLOS ONE does not copyedit accepted manuscripts, so the language in submitted articles must be clear, correct, and unambiguous. Any typographical or grammatical errors should be corrected at revision, so please note any specific errors here.

Reviewer #1: Yes

Reviewer #2: Yes

**********

5. Review Comments to the Author

Please use the space provided to explain your answers to the questions above. You may also include additional comments for the author, including concerns about dual publication, research ethics, or publication ethics. (Please upload your review as an attachment if it exceeds 20,000 characters)

Reviewer #1: Mohan & Biro present an experimental study of convention formation, manipulating visual access to the partner’s screen, whether participants were informed about the reward structure (i.e., both selecting the same color), and testing how conventions from one pair can get passed onto a new generation.

I co-reviewed this paper together with one of my Postdocs, and we both found it to be a very enjoyable experience. The introduction was very well written and did a great job of clarifying terminology and in summarizing the related literature. The research question was also very well put, and the hypotheses were appropriate and justified, even if they didn’t always work out.

Together, we have one suggestion for a theoretical explanation that could explain the results, one formatting suggestion that could improve readability, and then a small list of minor comments.

*Theoretical explanation for why task information made it harder for conventions to form*

One relevant theoretical perspective could be from Roberts & Goldstone (PLOS ONE 2011), where they show that having too much reactivity prevented groups from converging on a correct solution. Their task is framed as dinner guests figuring out how much to contribute towards a bill, with feedback being binarized as correct vs. incorrect. Having too much reactivity in the group actually made it harder to reach the solution, since the group would over-correct and vacillate between too high and too little. A similar phenomena could also explain the current results, since both individuals being informed of the goals could be trying to signal a convention, leading to over-correction. Thus, in this dyadic setting, you might need a leader-follower dynamic for conventions to converge, with a more active leader and a more passive follower. Instead, the informed condition could lead to unstable k-level reasoning that ends up hindering more than helping.

*Combining Methods:Analyses with results*

If the journal style allows, the Methods: Analyses section could be joined with results in order to make the details closer to where they become relevant. Currently, the formatting places a large distance between the description of methods and the context in which they are applied, making it hard to assess the appropriateness and also hard to interpret the results (if details have been forgotten). Additionally, this current formatting also places Figure 2 quite far from the results in which it presents.

*Minor comments*

- Goldstone & Ashpoole (2004) could be a good reference for early work on convention formation (~ pg. 3)

- Line 69: Roger’s paradox is probably not the best example in the context of conventions since it explores the hypothesis that culture emerged from natural origins

- Line 149: more consistency could be used when referring to the instruction type (before it was referred to as informed vs. uninformed)

- Lines 184-186: A subordinate clause would be appreciated to indicate what should be avoided by randomizing colors

- Could participants talk to one another during the 5 minute break?

- Were there different color sets across sessions? Could representations from one session bleed over to the next?

- How long was the gap between Phase I and Phase II? Could there be difficulty in remembering the conventions from Phase I?

- Figure 2 could benefit from titles to make panels A-C more self-evident. The current placement in the methods section also separates it quite a bit from the results section when it becomes relevant

- Overall performance from Sec 3.1 could be added as a plot

- While the current reaction time results didn’t work, what about testing within-dyad differences for pre vs. post convention formation? I suspect that this would grant greater power for such an analysis, although the effect of trial number would also have to be controlled for

- The justification to define stability of conventions emerging with at least 100 trials to analyze seems rather arbitrary

- Line 410: n = 19

- Line 411: n = 21

- Lines 545-551: Could this be explained by the arbitrariness of the task (i.e., is building a model about something arbitrarily debilitating)?

- Aplin et al., (Nature 2015) could also be a good reference for conventions in non-human animals, where they experimentally induced conventions in great tits that persisted through cultural transmission

-Lines 668-669: This could be understood as a mixed condition (I-NI). What could be the reason for the high speed of emerging conventions under this mixed condition, given the argument that instructions slowed down the emergence of conventions in Phase I?

- The know-how vs. know-what section in the discussion is currently missing references. Maybe Bandini et al., (2020) is a relevant reference in the context of social/cultural learning, but there are likely to be earlier uses of the term

Reviewer #2: I recommend minor revisions to this paper. I recommend a few related literature, comment on some revisions on the Experimental Design, and suggest adding information on Pre-registration. Please see the attached Report for more details.

**********

6. PLOS authors have the option to publish the peer review history of their article (what does this mean?). If published, this will include your full peer review and any attached files.

If you choose “no”, your identity will remain anonymous but your review may still be made public.

**Do you want your identity to be public for this peer review?**  For information about this choice, including consent withdrawal, please see our Privacy Policy.

Reviewer #1: No

Reviewer #2: No

**********

[NOTE: If reviewer comments were submitted as an attachment file, they will be attached to this email and accessible via the submission site. Please log into your account, locate the manuscript record, and check for the action link "View Attachments". If this link does not appear, there are no attachment files.]

To ensure your figures meet our technical requirements, please review our figure guidelines: https://journals.plos.org/plosone/s/figures

You may also use PLOS’s free figure tool, NAAS, to help you prepare publication quality figures: https://journals.plos.org/plosone/s/figures#loc-tools-for-figure-preparation.

NAAS will assess whether your figures meet our technical requirements by comparing each figure against our figure specifications.

Attachment Submitted filename: Referee_Report_PLOS One.pdf

# Author comment: Author response to Decision Letter 1

**DOI:** 10.1371/journal.pone.0341532.r002

19 May 2026

Please see the uploaded document (response_to_reviewers.pdf).

Attachment Submitted filename: response_to_reviewers.pdf

# Peer review: Decision Letter 1

**DOI:** 10.1371/journal.pone.0341532.r003

**Authors:** Caleb Cox

18 Jun 2026

PONE-D-26-01263R1

Experimental Emergence of Conventions in Human Dyads

PLOS One

Dear Dr. Mohan,

Thank you for submitting your manuscript to PLOS ONE. After careful consideration, we feel that it has merit but does not fully meet PLOS ONE’s publication criteria as it currently stands. Therefore, we invite you to submit a revised version of the manuscript that addresses the points raised during the review process.

Both reviewers are happy with the revised version and recommend acceptance. Reviewer 1 makes some additional minor comments that should be straightforward to address. Formally, this requires a minor revision, but I plan to simply simply evaluate the next revision myself with no need to send the paper back to reviewers.

Please submit your revised manuscript by Aug 02 2026 11:59PM. If you will need more time than this to complete your revisions, please reply to this message or contact the journal office at plosone@plos.org. When you're ready to submit your revision, log on to https://www.editorialmanager.com/pone/ and select the 'Submissions Needing Revision' folder to locate your manuscript file.

Please include the following items when submitting your revised manuscript:

- A letter that responds to each point raised by the academic editor and reviewer(s). You should upload this letter as a separate file labeled 'Response to Reviewers'.
- A marked-up copy of your manuscript that highlights changes made to the original version. You should upload this as a separate file labeled 'Revised Manuscript with Track Changes'.
- An unmarked version of your revised paper without tracked changes. You should upload this as a separate file labeled 'Manuscript'.

If you would like to make changes to your financial disclosure, please include your updated statement in your cover letter. Guidelines for resubmitting your figure files are available below the reviewer comments at the end of this letter.

If applicable, we recommend that you deposit your laboratory protocols in protocols.io to enhance the reproducibility of your results. Protocols.io assigns your protocol its own identifier (DOI) so that it can be cited independently in the future. For instructions see: https://journals.plos.org/plosone/s/submission-guidelines#loc-laboratory-protocols. Additionally, PLOS ONE offers an option for publishing peer-reviewed Lab Protocol articles, which describe protocols hosted on protocols.io. Read more information on sharing protocols at https://plos.org/protocols?utm_medium=editorial-email&utm_source=authorletters&utm_campaign=protocols.

As the corresponding author, your ORCID iD is verified in the submission system and will appear in the published article. PLOS supports the use of ORCID, and we encourage all coauthors to register for an ORCID iD and use it as well. Please encourage your coauthors to verify their ORCID iD within the submission system before final acceptance, as unverified ORCID iDs will not appear in the published article. *Only*  the individual author can complete the verification step; PLOS staff *cannot*  verify ORCID iDs on behalf of authors.

We look forward to receiving your revised manuscript.

Kind regards,

Caleb Cox

Academic Editor

PLOS One

Journal Requirements:

If the reviewer comments include a recommendation to cite specific previously published works, please review and evaluate these publications to determine whether they are relevant and should be cited. There is no requirement to cite these works unless the editor has indicated otherwise.

Please review your reference list to ensure that it is complete and correct. If you have cited papers that have been retracted, please include the rationale for doing so in the manuscript text, or remove these references and replace them with relevant current references. Any changes to the reference list should be mentioned in the rebuttal letter that accompanies your revised manuscript. If you need to cite a retracted article, indicate the article’s retracted status in the References list and also include a citation and full reference for the retraction notice.

[Note: HTML markup is below. Please do not edit.]

Reviewers' comments:

Reviewer’s Responses to Questions

**Comments to the Author**

1. If the authors have adequately addressed your comments raised in a previous round of review and you feel that this manuscript is now acceptable for publication, you may indicate that here to bypass the “Comments to the Author” section, enter your conflict of interest statement in the “Confidential to Editor” section, and submit your "Accept" recommendation.

Reviewer #1: All comments have been addressed

Reviewer #2: All comments have been addressed

**********

2. Is the manuscript technically sound, and do the data support the conclusions?

The manuscript must describe a technically sound piece of scientific research with data that supports the conclusions. Experiments must have been conducted rigorously, with appropriate controls, replication, and sample sizes. The conclusions must be drawn appropriately based on the data presented.

Reviewer #1: Yes

Reviewer #2: Yes

**********

3. Has the statistical analysis been performed appropriately and rigorously?

Reviewer #1: Yes

Reviewer #2: Yes

**********

4. Have the authors made all data underlying the findings in their manuscript fully available?

The PLOS Data policy requires authors to make all data underlying the findings described in their manuscript fully available without restriction, with rare exception (please refer to the Data Availability Statement in the manuscript PDF file). The data should be provided as part of the manuscript or its supporting information, or deposited to a public repository. For example, in addition to summary statistics, the data points behind means, medians and variance measures should be available. If there are restrictions on publicly sharing data—e.g. participant privacy or use of data from a third party—those must be specified.

Reviewer #1: Yes

Reviewer #2: Yes

**********

5. Is the manuscript presented in an intelligible fashion and written in standard English?

PLOS ONE does not copyedit accepted manuscripts, so the language in submitted articles must be clear, correct, and unambiguous. Any typographical or grammatical errors should be corrected at revision, so please note any specific errors here.

Reviewer #1: Yes

Reviewer #2: Yes

**********

6. Review Comments to the Author

Please use the space provided to explain your answers to the questions above. You may also include additional comments for the author, including concerns about dual publication, research ethics, or publication ethics. (Please upload your review as an attachment if it exceeds 20,000 characters)

Reviewer #1: I am grateful to the authors for addressing all of the previous comments in a thorough fashion. I have two very minor points I think can be addressed without needing to go through another round of review, which I describe below.

Line 126 "Open questions“: Among other things, the authors find that a) more information leads to slower convention formation probably due to over-correction from social feedback (line 667), and b) visual access leads to less stability of conventions most likely due to higher level reasoning (line 710). Given these results, is perhaps a more successful strategy to learn reward-contingencies rather than to view the task as a coordination game? The authors hint at this possibility in lines 679-700 by referring to similar interpretations from other studies. But since this is the opening question of the paper, this alternative interpretation could be highlighted more in the paper.

Lines 563-575: The authors correctly hypothesized that conventions formed in the opaque condition would be more stable. As a measure of stability they calculated the maintenance of Elo-ratings across 100 trials. However, stability could also be interpreted as a convention being stable after a change. The unexpected maintenance of conventions formed during the opaque condition (Session 1) after switching to the transparent condition (Session 2) could thus be interpreted as another indicator of stability.

Reviewer #2: I thank the author for addressing all comments carefully and professionally. I recommend accepting this paper. Green light now. Good job!

**********

7. PLOS authors have the option to publish the peer review history of their article (what does this mean?). If published, this will include your full peer review and any attached files.

If you choose “no”, your identity will remain anonymous but your review may still be made public.

**Do you want your identity to be public for this peer review?**  For information about this choice, including consent withdrawal, please see our Privacy Policy.

Reviewer #1: No

Reviewer #2: No

**********

[NOTE: If reviewer comments were submitted as an attachment file, they will be attached to this email and accessible via the submission site. Please log into your account, locate the manuscript record, and check for the action link "View Attachments". If this link does not appear, there are no attachment files.]

To ensure your figures meet our technical requirements, please review our figure guidelines: https://journals.plos.org/plosone/s/figures

You may also use PLOS’s free figure tool, NAAS, to help you prepare publication quality figures: https://journals.plos.org/plosone/s/figures#loc-tools-for-figure-preparation.

NAAS will assess whether your figures meet our technical requirements by comparing each figure against our figure specifications.

# Author comment: Author response to Decision Letter 2

**DOI:** 10.1371/journal.pone.0341532.r004

2 Jul 2026

We have uploaded a point-by-point response to the reviewer comments.

Attachment Submitted filename: response_to_reviewers_2.docx

# Editor report: Decision Letter 2

**DOI:** 10.1371/journal.pone.0341532.r005

**Authors:** Caleb Cox

7 Jul 2026

Experimental Emergence of Conventions in Human Dyads

PONE-D-26-01263R2

Dear Dr. Mohan,

We’re pleased to inform you that your manuscript has been judged scientifically suitable for publication and will be formally accepted for publication once it meets all outstanding technical requirements.

Within one week, you’ll receive an e-mail detailing the required amendments. When these have been addressed, you’ll receive a formal acceptance letter and your manuscript will be scheduled for publication.

An invoice will be generated when your article is formally accepted. Please note, if your institution has a publishing partnership with PLOS and your article meets the relevant criteria, all or part of your publication costs will be covered. Please make sure your user information is up-to-date by logging into Editorial Manager at Editorial Manager® and clicking the ‘Update My Information' link at the top of the page. For questions related to billing, please contact billing support.

If your institution or institutions have a press office, please notify them about your upcoming paper to help maximize its impact. If they’ll be preparing press materials, please inform our press team as soon as possible -- no later than 48 hours after receiving the formal acceptance. Your manuscript will remain under strict press embargo until 2 pm Eastern Time on the date of publication. For more information, please contact onepress@plos.org.

Kind regards,

Caleb Cox

Academic Editor

PLOS One

Additional Editor Comments (optional):

Reviewers' comments:

# Editor report: Acceptance letter

**DOI:** 10.1371/journal.pone.0341532.r006

**Authors:** Caleb Cox

PONE-D-26-01263R2

PLOS One

Dear Dr. Mohan,

I'm pleased to inform you that your manuscript has been deemed suitable for publication in PLOS One. Congratulations! Your manuscript is now being handed over to our production team.

At this stage, our production department will prepare your paper for publication. This includes ensuring the following:

* All references, tables, and figures are properly cited

* All relevant supporting information is included in the manuscript submission,

* There are no issues that prevent the paper from being properly typeset

You will receive further instructions from the production team, including instructions on how to review your proof when it is ready. Please keep in mind that we are working through a large volume of accepted articles, so please give us a few days to review your paper and let you know the next and final steps.

Lastly, if your institution or institutions have a press office, please let them know about your upcoming paper now to help maximize its impact. If they'll be preparing press materials, please inform our press team within the next 48 hours. Your manuscript will remain under strict press embargo until 2 pm Eastern Time on the date of publication. For more information, please contact onepress@plos.org.

You will receive an invoice from PLOS for your publication fee after your manuscript has reached the completed accept phase. If you receive an email requesting payment before acceptance or for any other service, this may be a phishing scheme. Learn how to identify phishing emails and protect your accounts at https://explore.plos.org/phishing.

If we can help with anything else, please email us at customercare@plos.org.

Thank you for submitting your work to PLOS ONE and supporting open access.

Kind regards,

PLOS ONE Editorial Office Staff

on behalf of

Dr. Caleb Cox

Academic Editor

PLOS One
